# Some American Hospitals

**Published:** 1892-02-20

**Authors:** 


### IX.—GARFIELD MEMORIAL HOSPITAL, WASHINGTON.

This is a small hospital, having about sixty beds, and is in course of extension. It is pleasantly situated. There is a most excellent view of the city from the roof. An old house was originally purchased which now forms the central or administration block, and pavilions have recently been built on either side of it, so that the plan has now become an example of the H-type. Three hundred and ninety patients were admitted during the year, of whom 61 were coloured, The hospital contains a number of private wards, to which patients are admitted upon payment of 25 dols. a-week, with such fees in addition for operations and medical attendance as may be mutually agreed to. The accommodation for pay patients is excellent in every way, and most inviting. Altogether, the Garfield Hospital is in excellent order, and shows what good work may be done by the ladies if they set their minds to do it; this institution being largely maintained by the Ladies' Aid Association. The hospital is under the superintendence of Miss S. F. Palmer, and its present condition reflects the greatest credit upon her, and shows that she is not only devoted, but most successful in maintaining discipline, good order, and thoroughness throughout the whole establishment. We congratulate the people of Washington upon the position of this small hospital, our visit to which gave us great pleasure, because everything appeared to be done so heartily and thoroughly.

### Training School for Nurses.

Nearly all the American hospitals, whether large or small, undertake the training of nurses, hold examinations, and issue certificates of graduation. Of course, the practice pursued differs in various points, and is in many points at variance with the rules enforced by the British hospital authorities. We propose to give certain articles on training in the "Nursing Mirror Supplement," and shall therefore reserve any remarks we have to make on the instruction of nurses in the United States for the present.

